# Hydrogen Peroxide and GA_3_ Levels Regulate the High Night Temperature Response in Pistils of Wheat (*Triticum aestivum* L.)

**DOI:** 10.3390/antiox12020342

**Published:** 2023-01-31

**Authors:** Purbali Mukherjee, Pavithra Suriyakumar, S. Vanchinathan, Veda Krishnan, Milan Kumar Lal, Prakash Kumar Jha, Viswanathan Chinnusamy, Anjali Anand, P. V. Vara Prasad

**Affiliations:** 1 Division of Plant Physiology, Indian Council of Agricultural Research (ICAR)-Indian Agricultural Research Institute, New Delhi 110012, India; 2 Division of Biochemistry, ICAR-Indian Agricultural Research Institute, New Delhi 110012, India; 3 Division of Crop Physiology, Biochemistry and Post-Harvest Technology, ICAR-Central Potato Research Institute, Shimla 171001, India; 4 Feed the Future Innovation Lab for Collaborative Research on Sustainable Intensification, Kansas State University, Manhattan, KS 66506, USA; 5 Department of Agronomy, Kansas State University, Manhattan, KS 66506, USA

**Keywords:** ABA, Glyceraldehyde-3-phosphate dehydrogenase, high night temperature, hydrogen peroxide, pistil, wheat, GA_3_

## Abstract

High night temperature (HNT) impairs crop productivity through the reproductive failure of gametes (pollen and pistil). Though female gametophyte (pistil) is an equal partner in the seed-set, the knowledge of the antioxidant system(s) and hormonal control of HNT tolerance or susceptibility of pistils is limited and lacking. The objectives of this study were to determine the antioxidant mechanism for homeostatic control of free radicals, and the involvement of abscisic acid (ABA) and gibberellic acid (GA_3_) in HNT stress protection in the wheat pistils of contrasting wheat genotypes. We hypothesized that HNT tolerance is attributed to the homeostatic control of reactive oxygen species (ROS) and hormonal readjustment in pistils of the tolerant genotype. The ears of two contrasting wheat genotypes—HD 2329 (susceptible) and Raj 3765 (tolerant) were subjected to two HNTs (+5 °C and +8 °C) over ambient, in the absence and presence of dimethylthiourea (DMTU), a chemical trap of hydrogen peroxide (H_2_O_2_). Results showed that HNTs significantly increased ROS in pistils of susceptible genotype HD 2329 to a relatively greater extent compared to tolerant genotype Raj 3765. The response was similar in the presence or absence of DMTU, but the H_2_O_2_ values were lower in the presence of DMTU. The ROS levels were balanced by increased activity of peroxidase under HNT to a greater extent in the tolerant genotype. Cytosolic glyceraldehyde-3-phosphate dehydrogenase (GAPC) activity was inversely related to H_2_O_2_ production within a critical range in Raj 3765, indicating its modulation by H_2_O_2_ levels as no change was observed at the transcriptional level. The hormonal status showed increased ABA and decreased GA_3_ contents with increasing temperature. Our study elucidates the role of H_2_O_2_ and GA_3_ in stress tolerance of pistils of tolerant genotype where GAPC acts as a ROS sensor due to H_2_O_2_-mediated decrease in its activity.

## 1. Introduction

Wheat (*Triticum aestivum* L.) is one of the world’s major cereal crops, with global production of 769.5 million tons in 2020–2021 [1]. It is consumed as a major source of calories through a variety of food products. Like many other food crops, wheat is susceptible to climate change, particularly increasing temperatures. Gourdji et al. [2] predicted that by 2030, around 11% of the global area occupied by wheat will experience high mean temperatures > 34 °C for at least five days during the reproductive period. A dissection of mean temperature into maximum and minimum temperatures has accentuated the occurrence of diurnal asymmetry between these temperatures [3]. Minimum temperatures have increased at twice the rate of maximum temperatures (0.204 °C per decade and 0.141 °C per decade, respectively) from 1950 to 2004 [4]. Furthermore, a seasonal variation in the wheat growing belt of India is also evident in the increment in minimum temperatures with 0.28 °C/decade during winter (October–March) and 0.19 °C/decade during autumn (October–November) [5]. This change would undoubtedly impact wheat production, as grain yield is reported to be strongly correlated with increasing minimum temperatures [6]. The vulnerability of reproductive stage to high temperature (HT) stress in most crops is attributed to abnormalities during the development of male and female gametophytes, pollen germination, fertilization, and embryo development [7,8,9]. Although research on heat-induced impairment of reproductive organs is limited to the male gametophyte [10,11], the indispensable participation of the female gametophyte in determining spikelet fertility cannot be overlooked [12,13,14,15,16,17]. Few studies have documented the susceptibility of the female gametophyte to HT stress, which is species-dependent [18,19]. The HT-sensitive response was associated with macropore degeneration in tomato (*Solanum lycopersicum* L.) [20], numerous stigma formations in rice (*Oryza sativa* L.) [21], shorter style, shrunken ovaries, higher number of ovules in chickpea (*Cicer arietinum* L.) [22], impairment of stigma and style in sorghum (*Sorghum bicolor* L. Moench) [23] and disintegration of nucleus and dehydration of cytoplasm of pistil in sorghum [17].

In wheat, HT stress diminished the nucellus and embryo sac [12], malformed and dried out the stigma, style, and ovary [15]. It was also observed that the germinated pollen tube could not receive a clear directional cue, resulting in its distortion and disrupted fertilization [18]. The callose deposition in ovaries was another reason for the prevention of pollen tube growth towards the ovule, causing sterility [16]. A recent review by Wang et al. [18] elucidated the differential response of pistil of various species to HT stress of varying degrees and intensities. Besides morphological and physiological effects of high day temperatures, research provided evidence that high night temperature (HNT) stress coinciding with anthesis in field-grown wheat and barley (*Hordeum vulgare* L.) reduced grain yield by 7% per °C increase in night temperature, due to the lower number of spikes per meter square [24]. In another study, HNT stress resulted in abnormal development of the ovule and poor ovule performance, which ultimately affected the development of the fertilized embryo sac in winter wheat [25]. The metabolic changes responsible for poor performance of the female gametophyte under HNT have, however remained elusive. The role of reactive oxygen species (ROS) in the developing male and female gametophyte under both normal and stressed conditions has been well established in a few crops. Higher ROS generation under HNT decreased pollen viability and germination due to membrane damage [15]. Zhang et al. [26] and Sharma and Bhatla [27] observed that ROS affect pollen tube growth inside the pistil of sweet cherry (*Prunus avium* L.) and sunflower (*Helianthus annus* L.), respectively. In a recent study, Shi et al. [28] found that pistils of a tolerant rice cultivar, Nagina-22, maintained ROS homeostasis and antioxidative enzyme balance during the post-stress recovery period. In addition, the ROS generated during HT stress can be perceived by Glyceraldehyde-3-phosphate dehydrogenase (GAPDH), a key enzyme in glycolysis that controls the energy production required for pollen germination [29].

Besides ROS, the endogenous levels of phytohormones like gibberellic acid (GA_3_) and abscisic acid (ABA) regulate the source–sink relationship in developing seed [30]. The levels of these hormones were altered in rice and wheat exposed to HT stress during the microspore stage, resulting in lower spikelet fertility [31]. The expression of genes involved in gibberellin and ABA biosynthesis was also altered, causing reduced and enhanced production, respectively [32]. However, the role of ROS and phytohormones in regulating the HNT response in pistil of wheat has not been investigated. Therefore, the objectives of this research were to (a) evaluate ROS management by the antioxidant system; and (b) the hormonal levels (ABA and GA_3_) associated with HNT response in pistils of contrasting wheat genotypes. We hypothesized that HNT tolerance may be imparted by homeostatic control of ROS and hormonal readjustment in pistils of the tolerant genotype. Keeping this in mind, we selected two contrasting wheat genotypes HD 2329 (sensitive) and Raj 3765 (tolerant) and exposed them to two intensities of HNT over the ambient night temperature (14 °C): HNT (+5 °C) and HNT (+8 °C) from 50% ear emergence until anthesis under controlled environmental conditions. In order to confirm the role of ROS in various metabolic processes, the experiment was carried out both in the absence and presence of the chemical trap of hydrogen peroxide (H_2_O_2_), Dimethyl Thiourea (DMTU). The different components of oxidative stress, like the concentration of ROS and their regulation through antioxidant enzyme activities, glycolytic enzyme activity, and cytosolic glyceraldehyde 3-phosphate dehydrogenase (GAPC) were studied under HNT stress. The role of ABA and GA_3_ under HNT stress was also investigated to unravel their contribution to the tolerance mechanism.

## 2. Materials and Methods

### 2.1. Experiment

#### 2.1.1. Treatment Details

Seeds from two contrasting wheat genotypes i.e., HD 2329 (heat sensitive) and Raj 3765 (heat tolerant) [33] were sown in Completely Randomized Design (CRD) in 8.5 L capacity pots (25.5 cm diameter × 24 cm height) filled with soil. A hundred pots were filled with 10 kg each of clean arable soil mixed with farmyard manure. Nitrogen was applied in the form of urea at the rate of 120 kg ha^−1^ as three split doses (50:25:25 of basal dose at the time of sowing, tillering, and heading stage, respectively). Phosphorus was applied as P_2_O_5_ in the form of single super phosphate @ 60 kg ha^−1^ and potassium as K_2_O in the form of muriate of potash @ 40 kg ha^−1^ at the time of sowing. Fifty pots per genotype were kept in rows under control (ambient) conditions until 50% ear emergence at the pot culture during 2021–2022.

#### 2.1.2. High-Temperature Treatments in Growth Chambers

**Figure 1:**
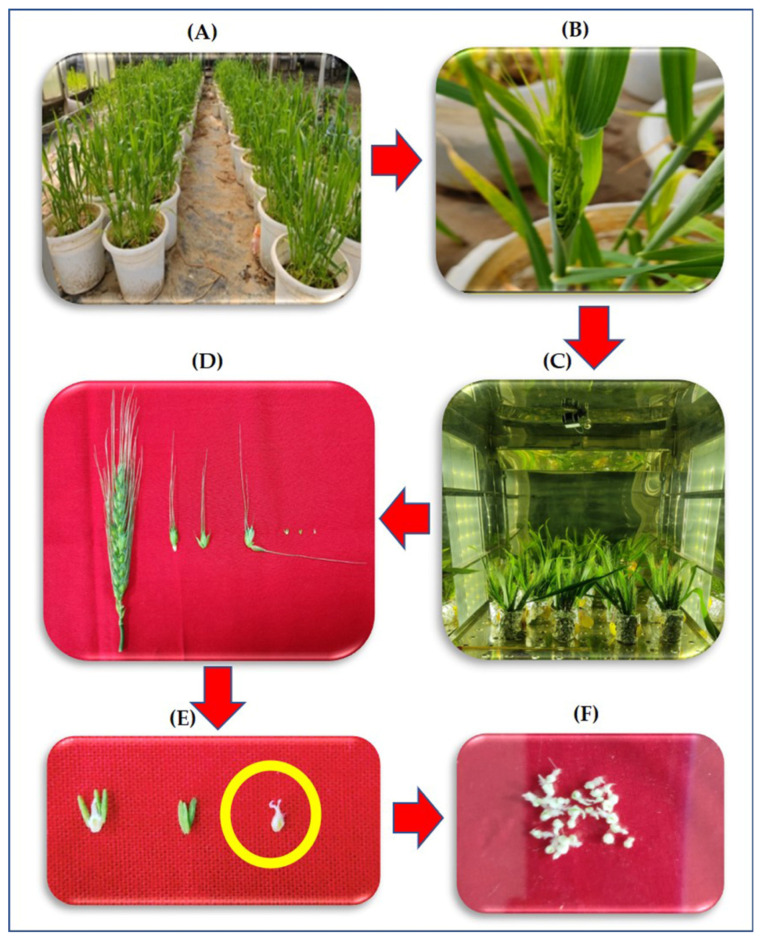
Flow diagram depicting the transfer of ear from plants to ear culture followed by sequential removal of pistil and collection of samples for study (**A**) growing of plants in the pot culture till 50% ear emergence, (**B**) Removal of the ear with intact flag leaf, (**C**) culturing of the ear in a growth chamber under different temperatures, (**D**) sequential removal of pistil from the central spikelet of the ear after four-night treatment of HNT, (**E**) enlarged image of the pistil, and (**F**) pooled pistils for various biochemical measurements.

Figure 1 illustrates the process of collection of the pistils from the ears for various biochemical measurements. The ears were collected in the evening between 5 p.m.–6 p.m. by keeping the flag leaf intact at 50% emergence stage in sets of five ears, which were cultured by immediately transferring them to flasks containing the media, as described by Singh and Jenner [34] (Table 1). The flasks were placed in three different chambers with night (6 p.m.–6 a.m.) temperatures maintained at +5 °C and +8 °C over ambient (14 °C) i.e., 19 °C and 22 °C, respectively. The daytime temperatures and humidity were similar at 27 °C and 65 ± 5%, respectively, for all treatments. Under each temperature treatment there were two sets of cultures: (1) without hydrogen peroxide (H_2_O_2_) chemical trap-DMTU [35] (2) and with 80 mM DMTU. The experimental design was Completely Randomized Design (CRD) with three factors (genotypes, temperatures, and DMTU). The hormonal analysis was done only in DMTU free media.

### 2.2. Histochemical Analysis of ROS in the Pistil

Spikelets were removed from the middle of the ear at the anthesis stage (4 nights of HNT) from all the treatments and were kept in 6 mM NBT (nitrobluetetrazolium) dissolved in 10 mM Tris HCl (trisaminomethane hydrochloride, pH 7.4) for three hours under dark conditions to detect superoxide radicals. The pistils were examined under a stereo microscope (Stemi 305 trino, White Plains, New York, USA) using T Capture software to obtain the image. Similarly, pistils were stained with 4.7 mM 3,3′-diaminobenzidine (DAB) prepared in 10 mM Tris HCl (pH 7.4) and used for detection of H_2_O_2_.

### 2.3. Quantification of ROS

Pooled pistils in triplicate from the two genotypes under all different treatments were frozen immediately in liquid nitrogen and kept in −80 °C for further biochemical analysis and quantification of various ROS.

#### 2.3.1. Superoxide Radicals

Pistil (40 mg) sample was homogenized in 1 mL of pre-cooled phosphate buffer (0.2 M, pH 7.2) by following the procedure given by Chaitanya and Naithani [36]. The homogenate was centrifuged at 10,000× *g* for 10 min at 4 °C. The supernatant thus obtained was used for estimation. To an aliquot (100 µL) of the above supernatant 3 mL reaction mixture containing 0.25 mM NBT, 25 mM sodium carbonate, 0.1 mM EDTA (Ethylene diamine tetra acetic acid), and 13.3 mM L-methionine was added. The entire reaction mixture was then incubated for a 10 min at 30 °C in a water bath. The absorbance was measured at 540 nm with a UV-visible spectrophotometer (Specord 210 Plus, Analytikjena, Jena, Germany).

#### 2.3.2. Hydrogen Peroxide

Pistils (40 mg) were ground in 1 mL of pre-cooled acetone and filtered. To the filtrate, 2 mL of titanium reagent and 2.5 mL of ammonium hydroxide solution was added and titanium-hydroperoxide complex was precipitated [37]. The mixture was centrifuged at 10,000× *g* for 10 min and dissolved in 5 mL of 2 M concentrated sulfuric acid and recentrifuged. The supernatant was collected and measured at 415 nm with a UV-visible spectrophotometer.

### 2.4. Antioxidant Enzyme Activity

#### 2.4.1. Extraction of Enzyme

Pistil tissue (40 mg) was crushed into a fine powder using liquid nitrogen in a chilled mortar and pestle by following the procedure of Dhindsa et al. [38]. In order to extract the soluble protein from the homogenized powder, 1 mL of a 50 mM potassium phosphate buffer (pH 7.0) containing 1 mM EDTA, 1% (*w*/*v*) PVP (polyvinylpyrrolidone), and 0.2 mM ascorbate was added. The homogenate was centrifuged at 10,000× *g* for 30 min at 4 °C. The supernatant (enzyme extract) was employed for the subsequent estimation of antioxidant activity with bovine serum albumin (BSA) was used as a reference to assess the total soluble protein [39].

#### 2.4.2. Determination of Superoxide Dismutase (SOD) Activity

A 3 mL reaction mixture was prepared containing 50 mM potassium phosphate buffer (pH 7.8), 13.3 mM methionine, 75 µM NBT, 2 µM riboflavin, 0.1 mM EDTA, 50 mM sodium carbonate and 0.1 mL enzyme extract [38]. The reaction mixture was illuminated for 15 min at 3600 flux light intensity in the growth chamber, and the absorbance read at 560 nm with a UV-visible spectrophotometer. The amount of enzyme needed to provide a 50% inhibition of NBT reduction was determined to be one unit of SOD. The specific activity was expressed as U mg^−1^ protein.

#### 2.4.3. Determination of Peroxidase (POX) Activity

The development of tetra guaiacol from guaiacol (extinction coefficient = 26.6 mM cm^−1^), was determined to estimate the peroxidase activity [40]. A 3 mL reaction mixture was prepared containing 50 mM phosphate buffer (pH 6.1), 16 mM guaiacol, 2 mM H_2_O_2_ and 20 µL of enzyme extract. The absorbance was measured every 30 s at 470 nm for 2 min with a UV-visible spectrophotometer.

#### 2.4.4. Determination of Catalase (CAT) Activity

Catalase activity was determined by following the consumption of H_2_O_2_ (extinction coefficient = 39.4 mM cm^−1^) by Aebi [41]. The 3 mL reaction mixture contained 50 mM phosphate buffer (pH 7.0), 10 mM H_2_O_2_ and 0.1 mL of enzyme extract. The absorbance was measured every 30 s for two minutes at 240 nm using a UV-visible spectrophotometer.

### 2.5. Estimation of Cytosolic Glyceraldehyde 3-Phosphate Dehydrogenase (GAPC) Activity

Pistil tissue (0.1 g) was homogenized in a chilled mortar and pestle using 600 µL of a solution containing 50 mM Tris-HCl (pH 8.0), 5 mM EDTA, 1 mM PMSF (phenylmethylsulfonyl fluoride), and 40 mM 2-mercaptoethanol in accordance with the method described by Eastmond et al. [42]. The homogenate was centrifuged at 12,000× *g* for 20 min at 4 °C. The supernatant was collected to assess GAPC activity by adding 50 µL of supernatant in a solution containing 50 mM triethanolamine- HCl (pH 8.0), 4 mM NAD+ (nicotinamide adenine dinucleotide), 10 mM sodium arsenate (Na_3_AsO_4_), 1.2 µmol of D-Glyeraldehyde-3-phosphate, 3 mM DTT along with distilled water to make the volume to 1 mL. The absorbance was measured every 30 s at 340 nm for 3 min using a UV-visible spectrophotometer. The activity of GAPC was calculated using the reduction of NAD+ (extinction coefficient = 6.22 mM cm^−1^).

### 2.6. Estimation of Gibberellic (GA_3_) and Abscisic Acid (ABA)

#### 2.6.1. Extraction of Hormones

Pistil sample (100 mg) was homogenized in 5 mL cold sodium phosphate buffer (0.05 M, pH 7.5) containing 0.02% sodium diethyldithiocarbamate (antioxidant) by following the procedure of Kelen et al. [43]. After collecting the homogenate, it was kept overnight at 4 °C in a shaker at 150 rpm. The sample was then centrifuged at 10,000× *g* rpm for 15 min at 4 °C followed by the collection of the supernatant. The samples were separated in a separating funnel using 2.5 mL of diethyl ether and the aqueous phase was collected and pH set at 2.5 using 1 N HCl. This was again partitioned two times using 2.5 mL of petroleum ether. Ether phase was discarded, and both the times aqueous phase was taken and further subjected to partitioning by adding 2.5 mL diethyl ether 3 times. At the last step, the ether phase was taken and filtered through sodium sulphate crystals using Whatman no. 1 filter paper. The filtrate was taken and kept for lyophilization, dissolved in 200 µL mobile phase (26:74; acetonitrile-HPLC water) for determination of GA_3_ and ABA.

#### 2.6.2. Quantification of Hormones

A calibration standard was prepared using GA_3_ and ABA in the range of 0.1–1.0 mg L^−1^. The HPLC (high performance liquid chromatography, Agilent 1100/1200 series, Agilent Technologies, Santa Clara, CA, USA) was loaded with the standards as a control with each run. The separation was accomplished on C18 column (Agilent Eclipse XDB-C18, 5 µm of pore size, 4.6 × 200 mm, Santa Clara, CA, USA) kept at 30 °C. The mobile phase was prepared by using 26% (*v*/*v*) acetonitrile prepared in HPLC grade water, filtered through PVDF (polyvinylidene difluoride) membrane filter (diameter: 13 mm, filtration rating: 0.45µm, Agilent Technologies, Santa Clara, CA, USA). The flow rate was maintained at 0.8 mL min^−1^ and 20 µL sample of extract was injected into the column. The wavelength for the determination of ABA and GA_3_ were 206 and 265 nm, respectively. The retention time for standards and hormones were monitored on the chromatograph. Using the ESTD (External Standard) quantification procedure, peak area was calculated to determine each hormone’s concentration. Following the same guidelines as before, the extracted samples and the external standard were calibrated and evaluated. It was compared to those of the calibration sample in order to determine the amount in the extracted sample.

### 2.7. Expression Analysis of Genes

#### 2.7.1. Total Ribonucleic Acid (RNA)and Complementary Deoxyribonucleic Acid (cDNA) Analysis

Three biological replicates of the pooled pistils from various temperature treatments were used to extract total RNA utilizing a modified Li & Trick [44] method. To get rid of proteins, the pistil sample (100 mg) was extracted in 500 µL of the extraction buffer containing 100 mM Tris (pH 9.0), 150 mM of sodium chloride (NaCl), 50 mM Na-EDTA, 1.5% SDS and 1.5% β-mercaptoethanol. The aforementioned extract was mixed vigorously before placing on the ice for 10 min. The phenol-chloroform mixture (1:1) was added (250 µL) to the extract after incubation and centrifuged at 13,000× *g* for 15 min at 4 °C. The upper aqueous phase was transferred to another Eppendorf tube containing 250 µL of Trizol reagent. By gently inverting the contents, the mixture was then incubated for 10 min at room temperature. Following incubation, 250 µL of chloroform-isoamyl alcohol (24:1) was added, and the mixture was then recentrifuged for 15 min at 13,000× *g* at 4 °C. The upper aqueous phase (around 350 µL) was transferred to the Eppendorf tube containing 100 µL of 2M NaCl. The contents were thoroughly mixed by inversion and stored at room temperature for 5 min. The RNA was precipitated using chilled isopropanol and then kept at −20 °C for 15 min. The sample was centrifuged at 13,000× *g* for 15 min at 4 °C, then the supernatant was discarded, and the RNA pellet was thoroughly cleaned with 500 µL of 70% ethanol. The RNA pellet was dried for 10 min at room temperature before being resuspended in a suitable amount of RNase free water. Agar-gel electrophoresis was used to confirm the integrity of the RNA after measuring its purity and concentration with a NanoDrop (Implen, Los Angeles, CA, USA). The RNA was kept at −80 °C until it was needed. Following the manufacturer’s instructions, the Verso cDNA Synthesis Kit (Thermo Fisher Scientific, Baltics UAB, Vilnius, Lithuania) was used to create first strand cDNA from 1 µg of RNA sample.

#### 2.7.2. Real Time qPCR Assay

Using the Primer Quest Tool of Integrated DNA Technologies (IDT Inc., Leuven, Belgium), a gene-specific primer pair for cytosolic glyceraldehyde 3-Phosphate dehydrogenase (*TaGAPC2*), ABA 8′-hydroxylase 1 (*TaCYP707A1*), ent-kaurene oxidase 2 (*TaKO2*), and *β-actin* (reference gene) was constructed on the exon-exon boundaries (Sigma-Aldrich, Mumbai, India) (Table 2). In all of the experiments, the target genes and actin were amplified simultaneously. Three biological replicates were used for each reaction, which was done in duplicate. The Bio-Rad CFX-96 Real-Time system was utilized to carry out the reactions. Thermocycling conditions included a hot start at 95 °C for 15 min, 39 cycles at 95 °C for 15 s, and 60 °C for 1 min. Following amplification, the existence of a single peak on the dissociation curve served as an indicator of the PCR (polymerase chain reaction) specificity. The primer efficiency of the reference and target genes was in the range of 92–114%. The 2^−∆∆CT^ method of Livak and Schmittgen [45] was used to determine the relative gene expression level between the control and HNT treated samples.

### 2.8. Statistical Analysis

Statistical analysis was conducted by one-way analysis of variance (ANOVA) using SPSS software package version 16.0 for Windows (SPSS Inc.) within treatments of each genotype independently. Duncan’s post hoc test at *p* ≤ 0.05 was performed with mean values to highlight the significant differences.

## 3. Results

There was significant influence on the biochemical traits related to antioxidant system at different temperature treatments for each genotype, in the presence and absence of DMTU.

### 3.1. ROS and Antioxidative Enzyme Activity

**Figure 2:**
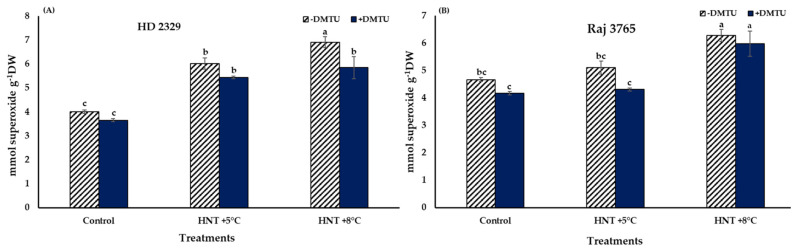
Effect of HNT on superoxide radical content in pistil of wheat genotypes (**A**) HD 2329 (sensitive) and (**B**) Raj 3765 (tolerant) under control, HNT (+5 °C) and HNT (+8 °C) conditions in absence and presence of ROS scavenger (DMTU). Bars represent mean ± SE. Bars with common letter are not significantly different between temperature treatments in a genotype at *p* < 0.05.

**Figure 3:**
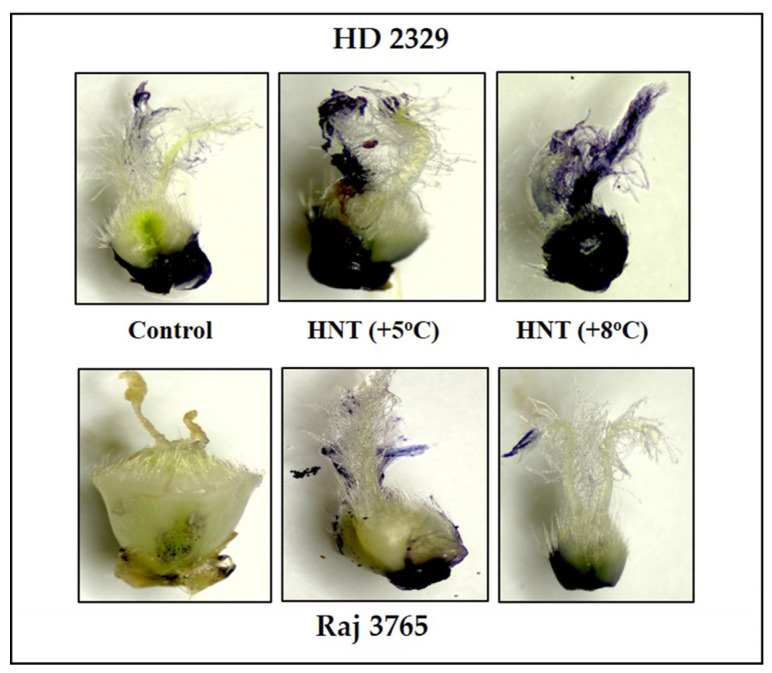
Photomicrographs showing superoxide anion (NBT staining) of pistil in HD2329 (sensitive) and Raj 3765 (tolerant) at control, HNT (+5 °C) and HNT (+8 °C) treatment.

Superoxide (O_2_^−.^) in the pistil of the flowers of the sensitive and tolerant genotypes growing under different temperatures varied with or without DMTU (Figure 2). In absence of DTMU, the pistil of the sensitive genotype (HD 2329) showed significantly higher (50.3 and 72.4% at HNT +5 °C and +8 °C, respectively) accumulation of O_2_^−.^ ions under both the HNT stress conditions, when compared to the control (Figure 2A). The photomicrographs of pistil stained with NBT also showed higher intensity of stain with increasing temperature (Figure 3). The addition of DMTU to the ear culture medium caused a significant increase (49.1%) at HNT +5 °C compared to ambient control. However, the values in presence of DMTU were reduced by 9.6 and 15.3% at HNT +5 °C and +8 °C, respectively, compared to their respective values without it (Figure 2A).

High night temperatures did not cause a significant increase in O_2_^−.^ levels in the tolerant genotype (Raj 3765) at HNT +5 °C but a 34.5% increase was evident at HNT +8 °C in the absence of DMTU (Figure 2B) which was also confirmed from the photomicrographs (Figure 3). The addition of DMTU caused a significant increase only at +8 °C compared to its ambient control (Figure 2B).

**Figure 4:**
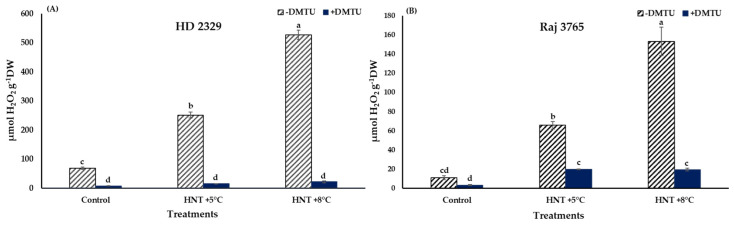
Effect of HNT on hydrogen peroxide content in pistil of wheat genotypes (**A**) HD 2329 (sensitive) and (**B**) Raj 3765 (tolerant) under control, HNT (+5 °C) and HNT (+8 °C) conditions in absence and presence of ROS scavenger (DMTU). Bars represent mean ± SE. Bars with common letter are not significantly different between temperature treatments in a genotype at *p* < 0.05.

A significant increase in H_2_O_2_ production was noted in HNT +5 °C and HNT +8 °C compared to control in HD 2329 (3.6- and 7.6-fold, respectively) in the absence of DMTU. The presence of DMTU resulted in 1.8- and 2.6-fold decrease in H_2_O_2_ content at the two elevated temperatures in comparison to ambient control. Overall, the H_2_O_2_ content decreased by 8.0–23.3-fold in the presence of DMTU compared to their respective values at each temperature (Figure 4A).

The increase in Raj 3765 was also to the tune of 6.1- and 14.2- fold higher under HNT +5 °C and +8 °C treatments, though the actual values in the tolerant genotype were 71–84% less than the susceptible genotype (Figure 4B). The addition of DMTU caused approx. 6.0- fold increase at the elevated night temperatures over ambient control. In addition, in this genotype, the H_2_O_2_ levels were reduced in the range of 3.3–7.9-fold with DMTU compared to their counterpart values at same temperatures (Figure 4B).

**Figure 5:**
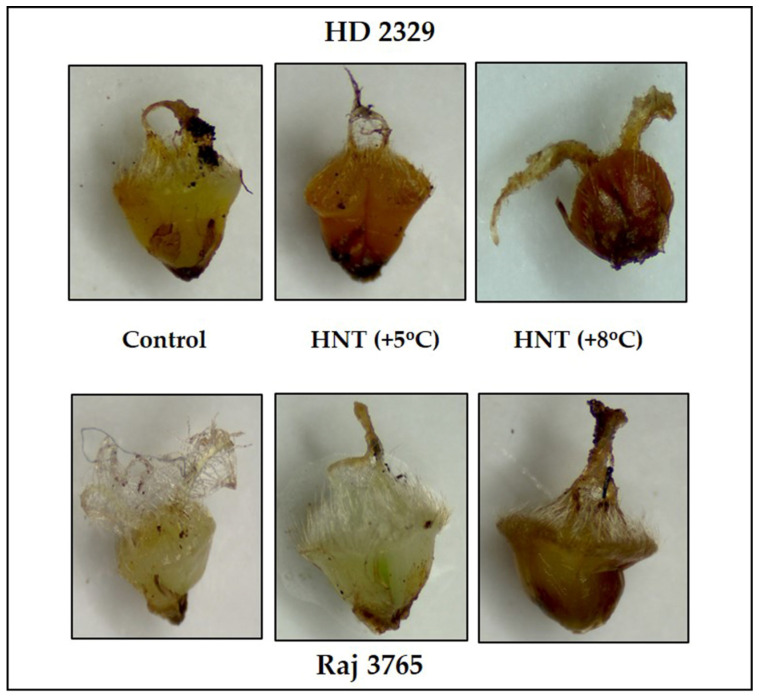
Photomicrographs showing hydrogen peroxide (DAB staining) of pistil in HD 2329 (sensitive) and Raj 3765 (tolerant) at control, HNT (+5 °C) and HNT (+8 °C) treatments.

Diaminobenzidine (DAB) staining of pistil also gave a clear indication and conformed to the quantitative measurements (Figure 5). The susceptible genotype showed pistil with increasing intensity of stain along with HNT. The tolerant genotype showed much less stain in control which was substantially higher at HNT +8 °C.

**Figure 6:**
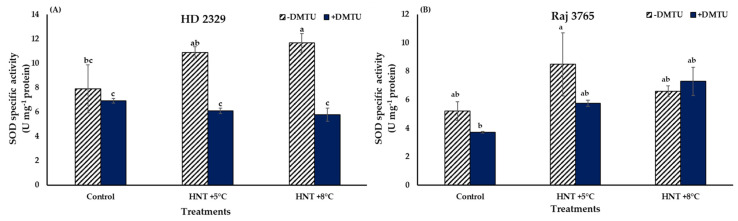
Effect of HNT on superoxide dismutase specific activity in pistil of wheat genotypes (**A**) HD 2329 (sensitive) and (**B**) Raj 3765 (tolerant) under control, HNT (+5 °C) and HNT (+8 °C) conditions in absence and presence of ROS scavenger (DMTU). Bars represent mean ± SE. Bars with common letter are not significantly different between temperature treatments in a genotype at *p* < 0.05.

The comparison between SOD specific activities at different HNT treatments of HD 2329 showed no significant difference between both the elevated temperature treatments compared to ambient control. With the addition of DMTU, the activities did not vary amongst the different temperature treatments, though the presence of DMTU led to 43 and 50% decline in activities at HNT +5 °C and HNT +8 °C compared to their values in its absence (Figure 6A).

The SOD activities remained similar at all temperature treatments in Raj 3765 with or without the presence of DMTU (Figure 6B).

**Figure 7:**
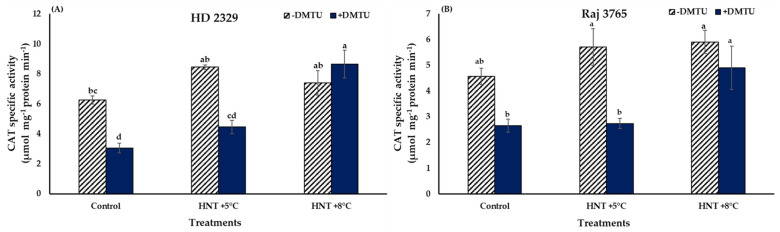
Effect of HNT on catalase specific activity in pistil of wheat genotypes (**A**) HD 2329 (sensitive) and (**B**) Raj 3765 (tolerant) under control, HNT (+5 °C) and HNT (+8 °C) conditions in absence and presence of ROS scavenger (DMTU). Bars represent mean ± SE. Bars with common letter are not significantly different between temperature treatments in a genotype at *p* < 0.05.

Catalase specific activity did not show a significant variation in both the genotypes under all the temperature treatments in absence of DMTU (Figure 7A,B). The presence of DMTU caused a 2.8- fold increase in activity in the pistil of susceptible genotype HD 2329 at HNT +8 °C temperature in comparison to its ambient control though the actual values were reduced in control and HNT +5 °C by approx. 50%. Similarly, the tolerant genotype Raj 3765 also showed a significant 1.9-fold increase in activity at HNT +8 °C temperature (Figure 7B).

**Figure 8:**
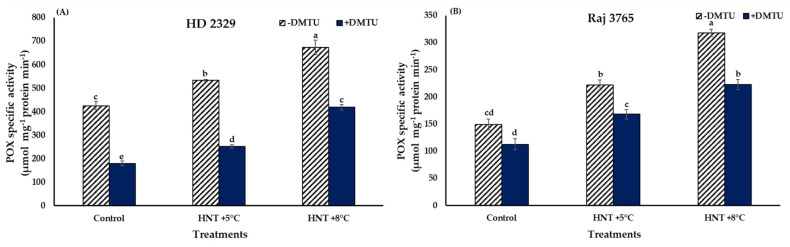
Effect of HNT on peroxidase specific activity in pistil of wheat genotypes (**A**) HD 2329 (sensitive) and (**B**) Raj 3765 (tolerant) under control, HNT (+5 °C) and HNT (+8 °C) conditions in absence and presence of ROS scavenger (DMTU). Bars represent mean ± SE. Bars with common letter are not significantly different between temperature treatments in a genotype at *p* < 0.05.

The trend in POX activity was similar to that observed for H_2_O_2_ in both the genotypes (Figure 8A,B). HD 2329 recorded significantly higher (25.4 and 58.6%) activity at HNT +5 °C and +8 °C over the ambient control in the absence of DMTU. The POX activity declined with the addition of DMTU, though the values were 1.4- and 2.3-fold higher at elevated temperatures in comparison to the ambient (Figure 8A). The addition of scavenger led to 37.9–57.8% reduction in the POX activity at all temperature treatments when compared to their activity without DMTU at the same temperature.

The increment in POX activity in Raj 3765 was similar to the pattern observed for H_2_O_2_ content, as 48.8 and 113.4% increase was observed at HNT +5 °C and HNT +8 °C, respectively, over ambient control, in the absence of DMTU (Figure 8B). The addition of DMTU resulted in 1.5- and 1.9-fold higher values at HNT +5 °C and HNT +8 °C in comparison to ambient temperature, which was similar to the sensitive genotype, HD 2329. However, there was a reduction of 24.2–29.9% in POX activity in presence of DMTU against its absence at all the temperature treatments (Figure 8B).

### 3.2. Cytosolic Glyceraldehyde 3-Phosphate Dehydrogenase (GAPC) Activity

**Figure 9:**
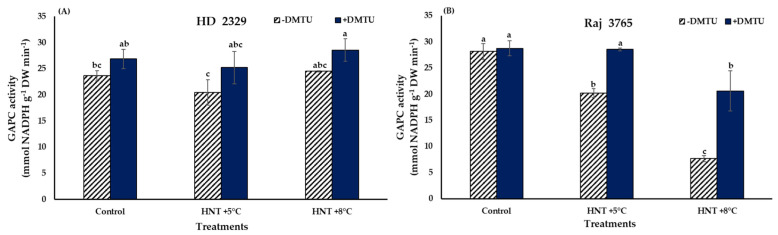
Effect of HNT on GAPC activity in pistil of wheat genotypes (**A**) HD 2329 (sensitive) and (**B**) Raj 3765 (tolerant) under control, HNT (+5 °C) and HNT (+8 °C) conditions in absence and presence of ROS scavenger (DMTU). Bars represent mean ± SE. Bars with common letter are not significantly different between temperature treatments in a genotype at *p* < 0.05.

The specific activity of GAPC was not affected significantly in susceptible genotype HD 2329 irrespective of the temperature treatments and the presence/absence of DMTU (Figure 9A). The tolerant genotype Raj 3765 showed a significant (28 and 72.7%, respectively) decline in GAPC activity compared to control under HNT +5 °C and HNT +8 °C, respectively, when DMTU was absent (Figure 9B). Addition of DMTU led to a significantly lesser (28%) activity only at HNT +8 °C.

**Figure 10:**
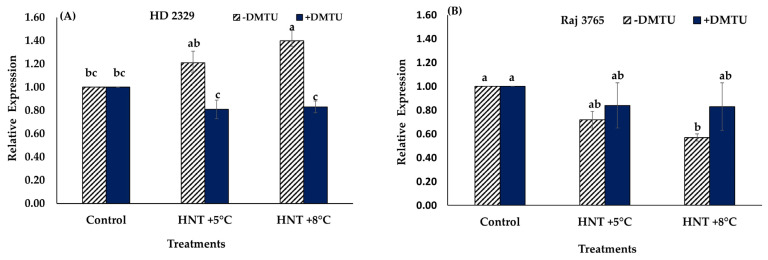
Effect of HNT on relative expression of *TaGAPC2* gene in pistil of wheat genotypes HD 2329 (sensitive) and Raj 3765 (tolerant) under control, HNT (+5 °C) and HNT (+8 °C) conditions in absence (**A**) and presence (**B**) of ROS scavenger (DMTU). Bars represent mean ± SE. Bars with the common letter are not significantly different between temperature treatments in a genotype at *p* < 0.05.

Expression analysis of *TaGAPC2* gene showed no significant difference in the relative expression of susceptible genotype HD 2329 under HNT +5 °C though it increased at +8 °C compared to control in absence of H_2_O_2_ scavenger (Figure 10A). Presence of DMTU did not affect the expression of the gene in this genotype (Figure 10A). However, tolerant genotype Raj 3765 showed a significant decrease in relative expression of *TaGAPC2* only under HNT +8 °C without DMTU in the medium (Figure 10B).

### 3.3. ABA and GA_3_ Concentration in the Pistils

**Figure 11:**
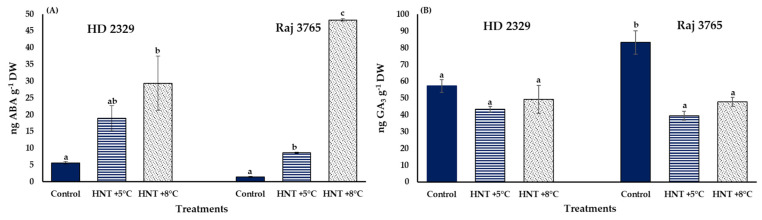
Effect of HNT on the concentration of (**A**) ABA and (**B**) GA_3_in the pistils of wheat genotypes HD 2329 (sensitive) and Raj 3765 (tolerant) under control, HNT (+5 °C) and HNT (+8 °C) conditions. Bars represent mean ± SE. Bars with common letter are not significantly different between temperature treatments in a genotype at *p* < 0.05.

A significantly high accumulation of ABA was observed for both the genotypes under HNT stress. It was increased by 3.3 and 5.2-folds in susceptible genotype HD 2329, and 6.0- and 33.7-fold in tolerant genotype Raj 3765 under +5 °C and +8 °C increase in HNT (Figure 11A). Interestingly, GA_3_ levels were stable across treatments in HD 2329 but decreased significantly by 47% at HNT +5 °C and 57% at HNT +8 °C in Raj 3765 (Figure 11B).

**Figure 12:**
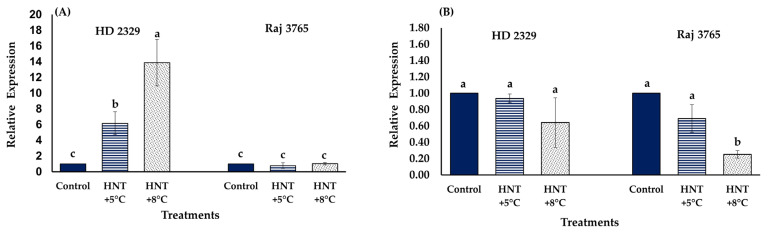
Effect of HNT on relative expression of (**A**) *ABA 8′-hydroxylase 1* (*TaCYP707A1*) and (**B**) *ent-kaurene oxidase 2* (*TaKO2*) gene in pistil of wheat genotypes HD 2329 (sensitive) and Raj 3765 (tolerant) under control, HNT (+5 °C) and HNT (+8 °C) conditions. Bars represent mean ± SE. Bars with common letter are not significantly different between temperature treatments in a genotype at *p* < 0.05.

The relative expression analysis of *TaCYP707A1* gene that encodes ABA 8′-hydroxylase, involved in ABA catabolism, showed no significant difference amongst different temperature treatments in Raj 3765, but increased significantly at HNT +5 °C (6.2-fold) and HNT +8 °C (13.9-fold) in HD 2329 (Figure 12A). The relative expression analysis of *TaKO2 gene* that encodes ent-kaurene oxidase (involved in GA_3_ synthesis) showed reduction in expression at HNT +5 °C (1.45-fold) and HNT +8 °C (3.96-fold) only in Raj 3765 (Figure 12B) while no change was observed in HD 2329.

## 4. Discussion

### ROS Production and Homeostasis under HNT

Environmental stresses like HT results in the generation of ROS viz. superoxide (O_2_^−^·), hydrogen peroxide (H_2_O_2_) and hydroxyl free radical (·OH) that are cytotoxic at high concentration and cause cellular damage through lipid peroxidation in the membranes, protein oxidation, damage to nucleic acids and activation of the pathway leading to programmed cell death [46,47]. In our research, the HT-sensitive genotype HD 2329 recorded relatively higher ROS at HNT compared to the tolerant genotype Raj 3765. Histochemical studies also complemented this observation. The trend remained similar after the addition of DMTU, as it is a scavenger of H_2_O_2_, and did not affect the levels of superoxide at all the temperature treatments. The H_2_O_2_ is a more stable and moderately reactive form of ROS. It is produced as a result of univalent reduction and protonation of O_2_^−^· [48]. The production of H_2_O_2_ increased with rising HNT in both genotypes, although the magnitude was markedly high in susceptible genotype HD 2329. Likewise, the photomicrographs of pistils showed more intense DAB stain with increasing HNT in susceptible HD 2329. While the tolerant genotype Raj 3765 showed a faintly stained organ with increasing intensity of night temperature. Our results conform to the findings in pearl millet (*Pennisetum glaucum* L. R.Br.) where temperatures above 38/28 °C resulted in high ROS levels in the pistils and spikelets [49]. Therefore, the role of ROS as stress indicators and secondary messengers for the regulation of antioxidant/defense genes during the stress response is unequivocal [50].

The steady-state level of ROS in the female gametophyte pistil is important for a successful seed set and plant function under stress conditions. The balanced levels are maintained by the enzymatic reactions catalyzed by superoxide dismutase, catalase, peroxidase, and non-enzymatic compounds like ascorbate, glutathione, and tocopherol [50,51]. Superoxide dismutase provides the first line of defense by reducing O_2_^−.^ to H_2_O_2_ and O_2_. We did not find a significant difference in the activity of SOD under different treatments for both the genotypes. This indicated that elevated levels of H_2_O_2_ under HNT stress were not the byproduct of the SOD enzymatic activity. Increased H_2_O_2_ levels may have been produced from other enzymatic reactions like plasma-membrane-bound NADPH oxidases and cell-wall-bound peroxidases [52,53] or non-enzymatically by dismutation of O_2_^−.^ to H_2_O_2_ under low pH conditions [54]. The homeostatic control of the levels of H_2_O_2_ within the cells is brought about by enhanced activities of catalase (CAT) or peroxidase (POX). Nevertheless, CAT activity also did not show any variation with HNT treatment, but the activity of peroxidase enzyme increased with the severity of stress in a pattern similar to that of H_2_O_2_ production. The confirmation of the involvement of peroxidase in scavenging H_2_O_2_ was also drawn from the experiments conducted with/without DMTU. The magnitude of POX activity was reduced in susceptible genotype HD 2329 in the presence of H_2_O_2_ scavenger, as lower levels of H_2_O_2_ stimulated lower activity of POX, albeit maintaining the increasing pattern with increasing HNT stress. Narayanan et al. [25] reported increased ROS, decreased antioxidant capacity, and photosynthesis in leaves of susceptible winter wheat genotype Karl 92 at high daytime and/or night-time in comparison to the optimum temperature. The seed set, grain number per plant, and grain yield were also compromised in Karl 92 while the damage was relatively less in the tolerant genotype, Ventnor [25].

Glyceraldehyde-3-phosphatedehydrogenase (GAPDH) is another important enzyme with a pivotal role in various abiotic and biotic stresses [55,56]. It is a glycolytic enzyme that catalyzes the reversible conversion of glyceraldehyde-3-phosphate into 1,3-bisphosphoglycerate in the cytosol and plastid [57]. Our observations on the activity of GAPC in the presence and absence of H_2_O_2_ scavenger demonstrated an inverse relationship between GAPC activity and H_2_O_2_ levels (although within a critical range of H_2_O_2_) in DMTU exclusion medium for the tolerant genotype. Low levels of H_2_O_2_ (~60–150 µmol g^−1^ DW) decreased GAPC activity while high levels observed in HD 2329 were non-responsive under HNT. It is probable that in our study, the concentration of H_2_O_2_, as seen in genotype Raj 3765, was effective in targeting GAPC, which may have a bifunctional role outside glycolysis [58]. In vitro studies in *Arabidopsis* have shown the inactivation of GAPDH by H_2_O_2_ [59]. This redox sensitive enzyme undergoes modification in the cysteine residue, which is mediated by H_2_O_2_ signaling in the cells during oxidative stress leading to its inactivation [60,61]. The inactivation of GAPDH may lead to downregulation of glycolytic pathway and metabolic re-routing of glycolytic cycle intermediates [62,63]. The addition of DMTU which reduced H_2_O_2_ levels substantially resulted in the loss of this relationship validating a previous report where addition of glutathione (GSH), an antioxidant, caused the reversal of inhibition of GAPC activity by H_2_O_2_ [59].

The change in GAPC activity could not be explained at the transcriptional level due to similar level of expression of *TaGAPC2*, irrespective of DMTU and temperature treatments. Nevertheless, few studies have related overexpression of *OsGAPC3* to alleviation of salt toxicity by regulating H_2_O_2_ levels [55]. It has been demonstrated that *AtGAPC1* and *AtGAPC2* are involved in plant responses to heat, anaerobic environments, salt, and drought stress [55,64,65]. Thus, our study revealed that H_2_O_2_ modulates the activity directly or indirectly by oxidative modification of the enzyme. Further investigations are needed on various regulatory components involved in the dose dependent H_2_O_2_- mediated regulation of GAPC activity. Such research can generate detailed information about the involvement of GAPC as a ‘ROS sensor’ under HT stress response. The physiological range of H_2_O_2_ concentration will be an important determinant of protein oxidation in the cells, as higher doses have not shown any significant relationship.

The regulation of developmental processes by the phytohormones, ABA and GA occur antagonistically, affecting the physiological response through an array of signaling pathways [66]. The pistils of both the genotypes showed increased ABA concentration with elevated temperature. Genotype HD 2329 had higher levels until HNT +5 °C compared to Raj 3765, the latter showing a substantial increase at HNT +8 °C. It has been reported that an increase in ABA during HT is a function of heat shock factor (HSFA6b) which is a downstream regulator in the ABA signaling pathway in *Arabidopsis* [67]. The trend was opposite in the case of GA_3_ levels, with only genotype Raj 3765 responding to increased temperature by lowering the GA_3_ levels. The involvement of paclobutrazol, an inhibitor of GA_3_ synthesis, has been implicated in HT stress tolerance in barley (*Hordeum vulgare* L.) which was negated by treatment with GA_3_ [68]. Thus, the reduced levels of GA_3_ in Raj 3765 could be associated with HNT tolerance under elevated temperature. A reduction in GA_3_ levels under HT changes the ABA/GA_3_ balance, which is important for a favorable stress tolerance response. In order to delineate the transcriptional regulation of both the hormones under HNT stress, we analyzed the transcript levels of cytochrome P-450 mediated monooxygenases: *ent-kaurene oxidase 2-like* (*TaKO2*), involved in the biosynthesis of GA_3_ and *ABA 8′-hydroxylase 1-like* (*TaCYP707A1*), involved in catabolism of ABA. The reduced expression of *TaKO2* could explain the lower levels of GA_3_ at HNT in Raj 3765, which is important to impact tolerance. On the contrary, a detailed analysis of all the other genes involved in the biosynthetic pathway of ABA will help to decipher the rate limiting enzyme associated with higher biosynthesis of ABA under HNT. The increased *ABA 8′-hydroxylase 1-like* (*TaCYP707A1*) expression of HD 2329 supported the trend of observed ABA levels with increasing temperature, thereby indicating that the concentration of ABA may be the result of its synthesis and degradation at different temperatures.

We acknowledge that one of major limitations of our study is lack of direct comparison of female and male gametes in the same study, and the lack of data on the seed-set to show the direct impact of yield components. Nonetheless, this research fills the critical knowledge gap on the impact of HNT on pistils alone, which is equally responsible for seed-set in grain crops. Further research on these aspects will improve our understanding of the relative tolerance or susceptibility of gametes and underlying biochemical and physiological reasons.

## 5. Conclusions

Our study revealed the differential response of pistils of tolerant and susceptible genotypes to HNT stress. There were greater levels of ROS under HNT in both genotypes, but values were higher in the susceptible genotype. The homeostatic control of H_2_O_2_ under stress was better maintained in the tolerant genotype through the increased activity of peroxidase compared to the susceptible genotype. The response was similar in the presence or absence of DMTU, but the H_2_O_2_ values were lower in the presence of DMTU. The H_2_O_2_ levels showed a close association with reduced activity of GAPC activity under stress, indicating it as an ROS sensor. Lower levels of GA_3_ indicated the hormonal control of tolerance in the tolerant genotype. Our study elucidates the role of H_2_O_2_ and GA_3_ in the stress tolerance of pistils in the tolerant genotype, where GAPC acts as a ROS sensor due to H_2_O_2_-mediated decrease in its activity. Future studies with direct comparison of male and female gametes with measures on various biochemical and physiological functions and supporting data on seed-set and seed numbers will confirm impact at the whole plant level, and provide a better understanding of their relative role and function in the whole plant stress tolerance.

## Figures and Tables

**Table 1:** Composition of ear culture medium.

Composition	Concentration (gL^−1^)
MS media	1.47
2[N-morpholino] ethane sulphonic acid	0.5
Glutamine	0.8
Sucrose	1.5

**Table 2:** Primers used to check the expression of different genes.

Gene Name	Accession No.	Primer Sequence (5′ to 3′)
*TaGAPC2*	LOC123160238	F-GTGGTGTCAATGAGAAGGAATA R-ATGGACTGTGGTCATCAAAC
*TaKO2*	LOC123161689	F-ATGGTTGCTACAAGTGACTAC R-CAACGTATGGAAAGTGCTTAAC
*β-actin*	AB181991	F-CGACTCTGGTGATGGTGTGAG R-AGCAAGGTCCAAACGAAGGA
*TaCYP707A1*	LOC123137905	F-ACCAAGTACAGATGGTCCAC R-CAGGCTTTGTTCTTGTCCTTG

## Data Availability

The data sets generated for this study are available on request to the corresponding author.
